# Obesity and Metabolic Care of Children of South Asian Ethnicity in Western Society

**DOI:** 10.3390/children8060447

**Published:** 2021-05-25

**Authors:** Ramya Sivasubramanian, Sonali Malhotra, Angela K. Fitch, Vibha Singhal

**Affiliations:** 1 Division of Pediatric Endocrinology, Massachusetts General Hospital for Children, Harvard Medical School, Boston, MA 02114, USA; dr.ramya.janaki@gmail.com (R.S.); smalhotra1@mgh.harvard.edu (S.M.); 2 MGH Weight Center, Harvard Medical School, Boston, MA 02114, USA; afitch@mgh.harvard.edu

**Keywords:** South Asian, thin-fat phenotype, childhood obesity, body fat percentage, central adiposity, metabolic risks

## Abstract

South Asians constitute one-fourth of the world’s population and are distributed significantly in western countries. With exponentially growing numbers, childhood obesity is of global concern. Children of South Asian ancestry have a higher likelihood of developing obesity and associated metabolic risks. The validity of commonly used measures for quantifying adiposity and its impact on metabolic outcomes differ by race and ethnicity. In this review we aim to discuss the validity of body mass index (BMI) and other tools in screening for adiposity in South Asian children. We also discuss the prevalence of overweight and obesity amongst South Asian children in western countries and the differences in body fat percentage, adiposity distribution, and metabolic risks specific to these children compared to Caucasian children. South Asian children have a characteristic phenotype: lower lean mass and higher body fat percentage favoring central fat accumulation. Hence, BMI is a less reliable predictor of metabolic status in these children than it is for Caucasian children. Furthermore, the relatively lower birth weight and rapid growth acceleration in early childhood of South Asian children increase the risk of their developing cardiometabolic disorders at a younger age than that of Caucasians. We emphasize the need to use modified tools for assessment of adiposity, which take into consideration the ethnic differences and provide early and appropriate intervention to prevent obesity and its complications.

## 1. Introduction

Childhood obesity is considered one of the most significant public health challenges, with exponentially growing numbers globally [1,2]. There has been a ten-fold spike in prevalence of obesity among school-age children and adolescents worldwide over the last four decades. The World Health Organization (WHO) stated the prevalence of obesity at over 38 million in children under 5 years of age in 2017, showing that younger children are also not spared from obesity. Childhood overweight and obesity has increased similarly in both developed and low-middle income countries since 1980 [3]. The term South Asians (SAs) denotes the population with origins from countries of South Asia including India, Pakistan, Bangladesh, Sri Lanka, Nepal, Bhutan, Afghanistan, and Maldives. SA constitute 25% of the world’s population. This ethnic group is widely distributed in western countries such as UK, US, and Canada [4,5,6,7]. Prevalence of obesity amongst SA adults in the US was found to be 77.6%, which was the highest in comparison to all other immigrant groups [4]. Children of SA ethnicity show a higher likelihood of overweight and obesity and a more precipitous increase in its severity when compared to Caucasian children [5]. Prevalence of obesity amongst children in native SA countries varies between 2–9% [6]. Immigrants arriving to the US at a younger age (≤20 years) have a higher likelihood of overweight and obesity over time compared to immigrants who arrive at older ages [7]. Notably, a study amongst pediatric refugees after US immigration highlighted that mean body mass index (BMI) percentile significantly increased from the 47th percentile to 63rd percentile within 3 years of US arrival and SA ethnicity was particularly affected [8]. In this review we aim to summarize the available literature that assesses the validity of BMI and other tools in appropriately screening for adiposity in SA children. We also discuss the prevalence of overweight and obesity amongst SA children in western countries and the differences in body fat percentage (BFP), adiposity distribution, metabolic risks, and interventions specific to these children. This comparison with Caucasian children is important as Caucasians form the majority in western countries and most of the guidelines made and followed by the primary care physicians use Caucasian children as the reference. This comparison helps clinicians to understand the relative differences between the two groups.

## 2. Methods

For this narrative review, a comprehensive search through online databases that included PubMed, Cochrane, and Google scholars was performed. A combination of the following keywords was used: ‘pediatric obesity, ‘overweight’, ‘central adiposity’, ‘prevalence’, ‘nutrition transition’, ‘immigrant’, ‘metabolic syndrome’, ‘Caucasian’, ‘South Asian’, ‘India’, ‘Pakistan’, ‘Nepal’, ‘Bangladesh’, ‘Sri Lanka’, ‘Bhutan’, ‘Maldives’, and ‘Afghanistan’. Subsequently, the articles were screened for suitability by reading the abstract and full-text article if needed. To obtain additional data, a manual search was performed using the reference lists of selected articles.

## 3. Literature Review

### 3.1. BMI and BFP in SA Children Compared to Caucasian Children

#### 3.1.1. Validity of BMI in Evaluating Body Adiposity in SA

BMI does not often capture the variations in BFP seen in children of different ethnicities, hence necessitating vital discussions on the ethnicity-specific validity of BMI as a screening tool. Due to the multi-ethnic population of Mauritius, a study was performed in Mauritius to assess the validity of BMI in children of Caucasian and SA ethnicity, especially Indian. Body composition was measured using an isotopic deuterium (2H) dilution technique as reference. This study showed that BMI is race and gender dependent and, hence, not a great tool to categorize obesity in children across ethnicities [9]. Lowest sensitivity within the population was reported in girls of SA ethnicity compared to other ethnic groups in Mauritius. Indian children were noted to have 8% higher BFP than the value indicated by BMI predictions. By using the WHO-defined BMI classification of obesity (BMI-for-age z-score > 2 SD), 2 out of 3 Indian girls with more than 30% body fat (cut-off for high adiposity) missed the diagnosis of obesity. The proportion having excess body fat defined by BFP was two times higher than those predicted by the BMI criterion [9]. A study from the United Kingdom (UK) calculated ethnicity-specific BMI adjustments that were derived by pooling data from studies that used deuterium dilution to assess fat mass as a reference and then assessed the ethnic differences in the BMI–body fat mass association [10]. They reported a consistent underestimation of body fat by BMI in SAs and suggested a positive BMI adjustment of +1.12 kg/m^2^ in SA boys and +1.07 kg/m^2^ in SA girls between 4–12 years. (Table 1) [11]. This is similar to the poor validity of BMI to assess BFP for other ethnicities, especially evidenced in African/American ethnicity [12].

#### 3.1.2. Alternate Tools to Replace BMI to Predict Adiposity

BMI has consistently proven to be a poor measurement for adiposity assessment in SA children. Meanwhile, alternate screening tools can be considered in SA as well as other ethnicities. In a study comparing body composition in SA adolescents with other European adolescents in the UK, skin fold thickness (SFT) was measured at four sites to measure BFP: biceps, triceps, suprailiac, and subscapular. This was followed by dual-energy X-ray absorptiometry (DXA) to assess the validity of BFP estimated by SFT [15]. DXA helps assess body composition from a whole-body scan by providing measures of lean and fat body mass. BFP derived from SFT was in sync with BFP derived from DXA scans in both SA and European adolescents. This study implied that SFT might be useful as a screening tool in SA adolescents [15]. Another method of body fat assessment, bioelectric impendence analysis (BIA), utilizes the estimation of total body water to estimate body fat. The uncertainty of hydration level of fat free mass in children at different stages of maturation makes BIA a less valid tool in this age group across ethnicities [16]. Amongst SA children, the estimation of BFP by BIA was significantly lower than the BFP estimated by DXA in both boys and girls across all age groups [17]. This is similar to the findings in Caucasian children, indicating low precision of BIA to estimate BFP [18]. However, unlike the extensive literature comparing BMI as a screening tool in SA versus Caucasian children, there is not enough data comparing these groups on other BFP measurement techniques such as air-displacement plethysmography and underwater weighing. On the other hand, DXA has often proven to be an efficient and accurate technique to assess fat mass in children as well as infants, although it is associated with minimal radiation exposure across ethnicities. DXA is in fact considered the gold standard to measure body composition [19,20,21]. Mid-upper arm circumference (MUAC) has shown potential as a low cost, convenient, and reliable alternative to BMI to identify overweight and obesity in all pediatric age groups [22,23]. When it comes to global relevance of MUAC, a study across 12 countries and the socioeconomic spectrum, including an SA country, stated that MUAC as a tool is accurate for identifying obesity in 9–11-year-old children across the 12 countries. The study measured reference BFP in these children using a portable Tanita SC-240 scale, which has been found to be comparable to DXA [24].

#### 3.1.3. Prevalence of Overweight and Obesity in SA Children in Western Countries and the Impact of Using BMI Adjustments

Prevalence of obesity amongst children in native SA countries varies between 2–9% [6]. In England, compared to Caucasian children, SA children had a lower prevalence of overweight and obesity when using unadjusted BMI to categorize the 4–5-year-old age group and a higher prevalence in the 10–11-year-old age group (42% in boys, 34% in girls SA) compared to Caucasian children (23% in boys and 21% in girls). However, by applying the suggested BMI adjustments (as discussed in Section 3.1.1: positive BMI adjustment of +1.12 kg/m^2^ in SA boys and +1.07 kg/m^2^ in SA girls between 4–12 years) the overweight-obesity prevalence in SA children increased massively in the 4–5 years age group (39% in boys, 35% in girls) and in the 10–11 years age group (52% in boys, 44% in girls), surpassing the prevalence amongst Caucasian children in both age groups [11]. Similarly, a study in Norway that assessed BMI in preschool children from various ethnic groups, initially noted that SA children had a lower prevalence of overweight and a higher prevalence of underweight (defined as below 18.5 kg/m^2^) compared to other ethnic Europeans. However, once the new BMI adjustment of +1.12 kg/m^2^ was applied, the prevalence of overweight increased by three times (from 5.2% to 14.3%) in SA children and prevalence of underweight decreased steeply (from 26% to 3.9%) [13]. Similarly, analyzing the relationship between BMI and adiposity among 2-year-olds of different ethnic groups in New Zealand, it was revealed that despite having similar fat mass (assessed by BIA), Indian children had a lower BMI z-score compared to children of European ethnicity [14]. These studies unanimously conclude that BMI is not the best screening tool for predicting adiposity in SA children even in the youngest ages (Table 1).

#### 3.1.4. Differences in Adipose Tissue Distribution in Children of SA Ethnicity

Differences in visceral adiposity in SAs exist extremely early in life. A study that compared healthy SA, especially Indian, and Caucasian infants within two weeks of birth noted that visceral adipose tissue (VAT) as assessed by whole body magnetic resonance imaging was much higher in SA infants revealing that ethnic differences in body fat distribution are present at birth [25]. Healthy full-term Indian neonates with similar whole body fat mass on magnetic resonance imaging have higher visceral adiposity than do Caucasian babies (Table 2) [25].

Ethnic differences in subcutaneous adipose tissue (SAT) depots have also been documented. Children whose BFP was identified by DXA were then compared on their SFT as a tool to gauge their SAT. This study revealed that SA children had lower SFT compared to that of their Caucasian counterparts, even when they had higher BFP as documented by DXA [26]. This indicates that SAT is much lower in SA children compared to that of Caucasian children. A substantial proportion of body fat in Indian children is, therefore, located outside the subcutaneous compartment, contributing to high visceral adiposity (Table 2).

### 3.2. Unique Phenotype and Trends in Fat Accumulation in Children of SA Ethnicity

**Thin-Fat phenotype (TFP)**: TFP refers to a phenotype where individuals have lower lean mass and high BFP even when weight status is in a “healthy” range. This falsely captures a healthy weight status even in the setting of higher metabolic risk. SA ethnicity is a risk factor for this phenotype from early age; SA neonates in comparison with Caucasian neonates have lower birthweight, smaller abdominal viscera, and lower muscle mass but preserved body fat [25,28,29]. TFP persists in SA children during pre-pubertal years [26]. Evaluating trends in weight gain in children of Indian ethnicity demonstrated that, in boys, the BFP tends to increase from 7 years of age until 11 years. Subsequently, there is a trend of decrease in BFP reaching a plateau by 14 years. In girls, a progressive rise in BFP from the age of 7 years continues until 17 years when it reaches peak. These gender-specific BFP trajectories are similar to those of Caucasian children [30]. However, Indian children demonstrated a higher degree of body fat accumulation in their peripubertal years compared to that of their Caucasian counterparts [27]. Ethnic differences in body proportions specific to SAs included larger waist circumference, waist-hip ratio, and truncal fat [15]. Further, all children of SA ethnicity show higher susceptibility to obesity (Table 2) [31].

**Thrifty-genotype, thrifty-phenotype, and fetal insulin hypothesis:** Thrifty-genotype and fetal insulin hypothesis state that multi-generational malnutrition has led to in-utero programming such that neonates of SA ethnicity are well adapted to an environment that is nutritionally limited but are more likely to become unhealthy in a nutritionally rich environment as they tend to accumulate more fat. It is noted that neonates with the thrifty phenotype, when exposed to an obesogenic environment postnatally, tend to have a higher likelihood of developing obesity and insulin resistance [32,33]. Despite the smaller size at birth, by 5 years of age, children from SA and Caucasian ethnicities had similar weight and height measurements, indicating a trend of accelerated growth in early childhood in SAs [34]. It is suggested that such growth acceleration has strong association to the later onset of obesity and risks of cardiovascular disorders mediated by an increase in blood pressure, fat deposition, and insulin resistance [35]. SA children with lower lean mass and rapid weight gain increase their vulnerability to cardiometabolic disorders. This is because an increase in BMI in these children mainly represents an increase in fat tissue. Hence, providers must be aware that SAs are vulnerable to excess weight gain and pay close attention to weight trajectories during early childhood even before the children reach the overweight threshold [36].

**Parental obesity and differing traditional food choices:** Prevalence of overweight and obesity amongst SA adults in the US was found to be 77.6%, making SAs the immigrant minority with the highest prevalence of obesity in the US [4]. Transgenerational obesity risks mediated through maternal insulin resistance, genetics, or lifestyle should be factored in while screening SA children [37]. A transition out of traditional food choices and habits and adapting a more sedentary lifestyle in immigrant SA populations has been suggested as an explanation for the significant increase in the prevalence of obesity and other risk factors contributing to type 2 diabetes mellitus and cardiovascular diseases [38].

**Genetic variants underlying obesity risk in those of SA ethnicity:** Specific genetic variants in SAs could be contributing to their higher predisposition to accumulate visceral adipose tissue and their high metabolic risks. Over 750 genetic loci have been identified via genome-wide association studies; however, most of these studies were conducted in individuals of Caucasian ethnicity [39]. Studies have also pointed towards certain genetic variants such as variants of the FTO (fat mass and obesity associated) gene in SAs showing association to metabolic risks similar to Caucasians, but differing from Caucasians when these genetic variants in SAs lack association to BMI [40]. While this could imply underlying differences between SAs and Caucasians in the mechanisms linking body size with metabolic risks [40], in contrast, some studies demonstrate that there are not many different genetic variants in SAs that are attributable to the differences in adiposity distribution [41].

### 3.3. Variation in Risks of Metabolic Syndrome between Children of SA vs. Caucasian Ethnicity

**Figure 1:**
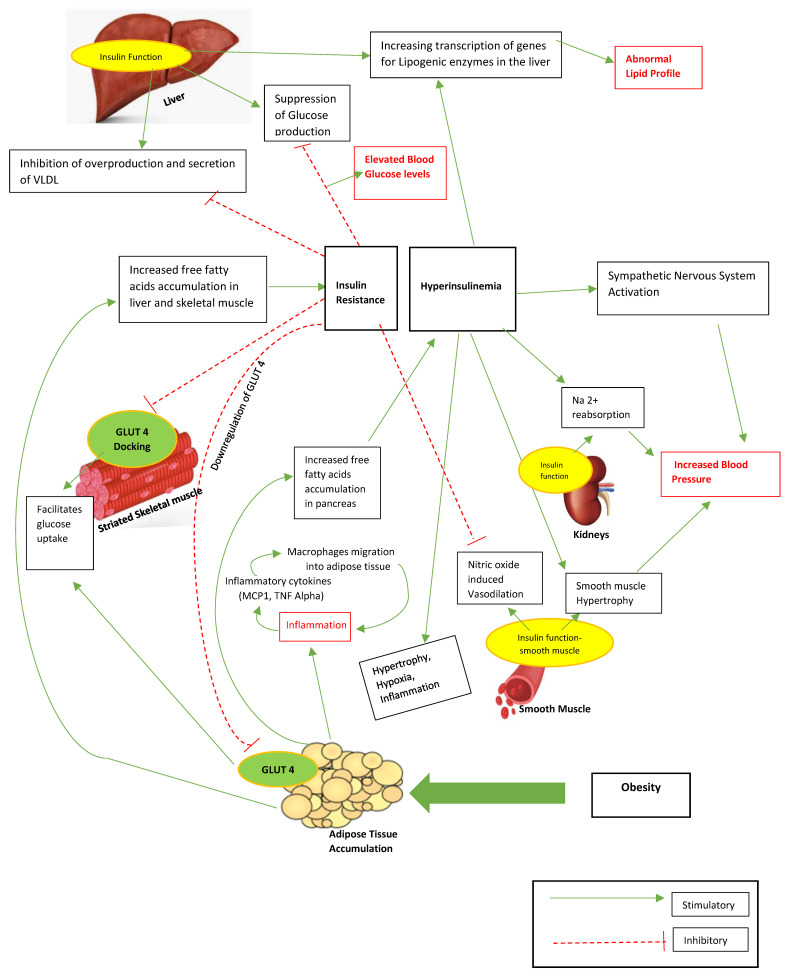
Obesity: insulin resistance model. This is an original illustration by the authors. VLDL: very low-density lipoprotein, GLUT 4: glucose transporter type 4 found on striated muscle and adipose tissue, Na^2+^: sodium, MCP1: monocyte chemoattractant protein 1, TNF Alpha: tumor necrosis factor alpha. The interaction between obesity, insulin resistance, and inflammation leads to metabolic syndrome. Excess free fatty acids deposit in the liver, adipocytes, skeletal muscles, and the pancreas, triggering impairment in insulin signaling and compromising the liver’s suppression of glucose production. Increased insulin levels stimulate lipogenic enzymes in the liver leading to increased production of triglyeride (TG). Insulin resistance and hyperinsulinemia can also lead to elevated blood pressure by causing 1. endothelial dysfunction, 2. insulin-induced sympathetic excitation, which leads to vasoconstriction and arterial hypertension, 3. sodium retention in kidneys, and 4. smooth muscle hypertrophy [42,43,44,45].

The interaction between obesity, insulin resistance, and inflammation may orchestrate the development of metabolic syndrome [42]. Obesity causes insulin resistance by the pathogenesis where excess free fatty acids deposit in the liver, adipocytes, skeletal muscles, and the pancreas, triggering impairment in insulin signaling [43]. This compromises the liver’s role in suppressing glucose production. Increased insulin levels stimulate lipogenic enzymes in the liver leading to increased production of triglycerides (TG). Insulin resistance and hyperinsulinemia can also lead to elevated blood pressure by leading to endothelial dysfunction [44] through insulin-induced sympathetic excitation. This in turn leads to increased cardiac output, vasoconstriction, and arterial hypertension [45] (due to insulin’s anabolic function of stimulating sodium retention in the kidneys) and induces smooth muscle hypertrophy (Figure 1) [42].

There has been an increase in the identification of the components of metabolic syndrome in the pediatric population, although currently there is no consensus on diagnostic criteria of metabolic syndrome in children. In adults, metabolic syndrome is defined as the presence of three of the following five criteria: central obesity, impaired glucose tolerance or presence of diabetes, hypertriglyceridemia, low high-density lipoprotein, and hypertension [46].

#### 3.3.1. Visceral Adiposity and Lean-NAFLD in SAs

Children and adolescents with higher visceral adiposity are more at risk of developing metabolic syndrome and non-alcoholic fatty liver disease (NAFLD) compared to those with higher subcutaneous adiposity [47]. Since, SA children have higher predisposition for visceral adiposity compared to Caucasian children, this may put them at a higher risk for these related comorbidities. Several adult studies have clearly highlighted the higher risk of NAFLD in SAs compared to that of Caucasians [48,49]. Pediatricians should be cognizant of the probable increased risk of NAFLD in SA children, even when they are within the recommended BMI range, as SAs present with NAFLD at a much lower BMI, known as lean-NAFLD in this population [50].

#### 3.3.2. Ethnic Differences in Systemic Inflammation

Comparing SA and Caucasian ethnicities for inflammatory markers, it was noted that SA infants (under 2 years of age) did not have elevated circulating markers compared with that of Caucasian children [51]. However, the higher levels of circulating IL-6 were noted in adult SA women, indicating higher systemic inflammation in SA women as compared to that of Caucasian women [52]. This study conducted in men and women who were SA or Caucasian showed higher systemic inflammation in SA women, who had 30% higher levels of the IL-6 than did Caucasian women. This was attributed to differences in body fat percentage (visceral and total) [52]. While these data are available only in adult women, it is likely to extrapolate to children. However, this needs investigation. This is an important finding because the potential link between abdominal obesity and the development of metabolic syndrome is inflammation mediated by alterations of adipokines such as adiponectin, leptin, tumor necrosis factor alpha (TNF-a), and interleukin-6 (IL-6), which are secreted by adipose tissue [53].

#### 3.3.3. Ethnic Differences in Insulin Resistance

Insulin resistance, studied in children of different ethnic groups after adjustment for adiposity and other confounders, is more prevalent among SA children compared to Caucasian children as early as 10 years of age [54]. Further, a study revealed that SA neonates have higher circulating insulin concentration than do Caucasian neonates after adjusting for differences in birthweight. This implies that the hyperinsulinemic, insulin-resistant phenotype of SAs is present at birth [55]. SA girls were noted to have increased fasting plasma glucose values compared to their Caucasian counterparts [56]. This translates to the highest prevalence of diabetes mellitus in SA adults in the US from among all immigrants [36]. The increased predisposition in SA children to high BFP, excess central adiposity, and systemic inflammation contributes to their higher risk of developing insulin resistance and metabolic syndrome compared to that of Caucasians (Table 3) [57].

#### 3.3.4. Ethnic Differences in Lipid Profile

A study analyzing adolescents of various ethnic groups in Canada suggested that abnormal lipids (high levels of low-density lipoprotein (LDL), low levels of high-density lipoprotein (HDL), or high levels of triglycerides (TG)) are more elevated in SA adolescents. It revealed that 62% of SA adolescents had at least one disordered feature of abnormal lipids whereas this was seen in only 49% of adolescents of Caucasian descent, despite no significant differences in dietary macronutrient intake among the ethnic groups. Abnormally decreased HDL levels and increased TG levels were noted in 54% and 30% of SA adolescents, respectively, indicating that insulin resistance is more prevalent in SA adolescents (Table 3) [56].

#### 3.3.5. Ethnic Differences in Blood Pressure and Cardiovascular disorders

SAs have demonstrated a higher prevalence of coronary artery disease, and can manifest this almost 10 years earlier than do other ethnic groups [58,59]. Childhood overweight and obesity can contribute to the development of premature atherosclerotic lesions, which ultimately leads to cardiovascular risks [62]. A study comparing SA children in Pakistan to Caucasian children in the US noted that SA children have a higher prevalence of elevated blood pressure (12.6%) compared to that of Caucasian children in the US (5%) despite lower BMI amongst SA children compared to that of Caucasian children [63]. There is evidence that for each 1–2 mm Hg increment of systolic BP during childhood, the risk of adult hypertension goes up by 10% [64,65]. Adolescents with risk factors for cardiovascular disease can transition into an adulthood with CVD [43,66]. Therefore, it is important to be aware of the higher predisposition to hypertension in SA children and adolescents compared to that of their Caucasian counterparts (Table 3).

#### 3.3.6. Ethnic Differences in Sleep Apnea

Although there is not enough literature comparing obstructive sleep apnea in SA children with Caucasian children, studies in adults have shown that SAs have a higher prevalence of OSA, a higher apnea-hypopnea index, and a higher severity of OSA compared to those of Caucasian adults [67].

## 4. Discussion

The growing prevalence of overweight and obesity and the higher predisposition to metabolic risks amongst SA children compared to that of Caucasian children in western countries is a serious concern. Evidence suggests underdiagnosis of obesity amongst SA children in western countries, making it vital for primary care providers to be aware of the unique ethnic aspects of screening and managing obesity in SA children. Higher visceral adiposity, lower subcutaneous fat, greater inflammation, and higher insulin resistance in SA children as compared to Caucasian children in the setting of near normal weight status make SA children particularly susceptible to metabolic adversity and poor health outcomes at a much younger age compared to Caucasians.

### 4.1. Poor outcomes in Adult Life: Obesity and Cardiometabolic Risks in SAs

There is evidence that compared to indigenous populations, immigrants from low- and middle-income countries have higher prevalence of cardiovascular diseases, and this is even higher in adults who immigrated at younger ages [68,69,70]. High risk for hypertension, T2DM, and mortality due to cardiovascular disease in adulthood is linked to low birth weight and rapid weight gain during infancy. This association must be kept in mind when monitoring SA children who have a higher likelihood of lower birth weight compared to Caucasian babies [71,72,73,74].

The presence of metabolic syndrome between the ages of 18–30 years is strongly associated with left ventricular dysfunction 5–10 years later [75]. In an ethnically diverse cohort in London, young adults of Pakistani and Bangladeshi origins were observed to have higher total cholesterol and lower levels of HDL than did their counterparts of other ethnicities. It was noticed that hemoglobin A1C (HbA1C) and HDL cholesterol levels in early adulthood were influenced by adiposity measures at early ages (11–13 years). Studies have also revealed that adverse effects of hyperglycemia on left ventricular function are more severe in SAs adults [60]. An explanation for the marked increase in these risks among SAs could be the probable epigenetic alterations in response to early life exposures in utero and during early childhood [76,77]. A three times higher prevalence of left ventricular hypertrophy and a greater degree of concentric remodeling are evident in SAs compared to that of Caucasian adults in Britain with heart failure manifesting at a much younger age in Indian immigrants [61]. Thus, it is important to identify and manage risk factors in the pediatric population to prevent complications during adult life (Table 3) [78].

### 4.2. Importance of Developing Ethnicity Sensitive Screening Tools

DXA is considered the gold standard to measure body composition [19], but because it is complex and expensive in children across ages and ethnicities, anthropometric measures such as body mass index (BMI), waist circumference, and waist-to-height ratio (WHR) have been used as alternatives [79]. However due to the BMI’s inability in accurately differentiate between fat mass and fat-free mass [80], it is a poor screening tool to estimate adiposity in SA children, as discussed in detail above. Pediatricians can consider using SA-ethnicity-specific adjustments as suggested by some studies (Section 3.1.1) to circumvent BMI associated underdiagnosis of obesity in SA children. Since there are not many studies that have derived BMI adjustments in SA children, it may be useful for future studies to replicate these findings to validate how much BMI underestimates BFP in SA children. Currently, the literature is lacking information on whether other body fat measurement techniques can be recommended over current practices for obesity screening in SA children in western countries. SA children and adolescents with obesity or overweight must be screened for type 2 diabetes mellitus in the presence of one of the following risk factors: signs of insulin resistance, maternal gestational diabetes during the child’s gestation, or family history of type 2 diabetes mellitus [81]. It is suggested that including waist measurements in routine pediatric screening can facilitate and improve future cardiometabolic risk stratification among children [82].

### 4.3. Recommendations to Tackle Childhood Obesity in Those of SA Ethnicity

See Table 4 and Table 5, family based dietary interventions are highly recommended as a primary management strategy to tackle overweight and obesity in children. Reduced energy diet, irrespective of macronutrient composition, is successful in managing a portion of children with overweight and obesity [83]. Disrupted mealtimes and increased consumption of fast food are major risk factors in SA children in the setting of urban migration [84]. Studies in children of Pakistani and Bangladeshi origins have shown that dietary choices that include fruits at least four times a week show a strong association with reduced obesity risk in SA children [85,86].

According to the 2018 WHO report, 81% of adolescents globally fall below the minimum recommended levels of physical activity [87]. Further, SA children display worse adherence to physical activity guidelines in comparison to that of Caucasian children [86,88]. There are several factors feeding into decreased physical activity in SA families: (1) inappropriate perception that a higher BMI indicates better health [89], (2) unfamiliarity with the concept of adequate physical activity, and (3) prioritizing time spent on work and education over physical activity amongst SA families in western countries [90].

Physical activity must be recommended strongly as part of the intervention plan to SA children with overweight or obesity, due to its ability to reverse insulin resistance in skeletal muscles and lower fasting insulin levels. Increased physical activity increases HDL and decreases LDL and TG concentrations, thereby improving the lipid profile. Systolic and diastolic blood pressures can also be lowered by the effect of exercise on improvement of endothelial function [91,92].

## 5. Conclusions

SA children have a higher BFP and lower lean mass compared to those of Caucasian children and display a high predisposition to excess central fat accumulation, hence, making BMI a poor adiposity predictor in these children. SA children have a lower birth weight and higher insulin resistance at birth compared to those of Caucasian babies and display sudden growth acceleration in early childhood. These features increase their vulnerability to cardiometabolic disorders at younger ages than Caucasians. When screening SA children for overweight and obesity these factors should be kept in mind. We need better tools for assessment of adiposity that takes into consideration the ethnic differences. Early identification will lead to earlier implementation of targeted therapies or prevention strategies that can decrease the risk of development of serious cardiovascular and metabolic disorders and the multitude of comorbidities associated with obesity.

## Figures and Tables

**Table 1:** BMI validity in evaluating BFP in SA children compared to that of Caucasian children.

Reference	Study Cohort	Findings	Body Fat Measurement Technique
Ramuth H et al. [9]	Mauritius: school children (200 boys and 177 girls, aged 7–13 years) of two main ethnic groups: Indian (SA) and Creole	BMI had the lowest sensitivity in Indian girls Indian children had ~8 % higher BFP than indicated by BMI predictions Using BMI, missed 2/3 Indian girls in Mauritius from being classified as having obesity	Isotopic deuterium dilution
Hudda M T et al. [11]	United Kingdom: based on the 2012–2013 National Child Measurement Program data in 582,899 children aged 4–5 years and 485,362 children aged 10–11 years	BMI consistently underestimated body fat in SAs Suggested a positive BMI adjustment of +1.12 kg/m^2^ for SA boys and +1.07 kg/m^2^ for SA girls across ages 4–12 years	Adjustments derived using deuterium dilution
Toftemo I et al. [13]	Norway children aged 4–5 years (n = 570) drawn from the population-based STORK Groruddalen cohort	Applying BMI adjustment of +1.12 kg/m^2^ in SAs increased the prevalence of overweight three-fold in SA children	Adjustments derived using deuterium dilution
Buksh MJ et al. [14]	Multi-ethnic cohort of 300 New Zealand children less than 2 years of age	Lower BMI z-score was found in Indian children compared to that of children of European ethnicity for similar fat mass	Body Impedence Analysis

**Table 2:** Trends in adiposity distribution seen in those of SA ethnicity.

References	Outcome	Findings
Modi N et al. [25]	Differences in adiposity amongst healthy SA versus Caucasian neonates as assessed by MRI	Higher visceral adiposity in SA neonates
Lakshmi S et al. [26]	Comparing SFT	Sum of four skin folds (biceps, triceps, subscapular, and suprailiac) in Caucasian children was higher than Indian children (+1.3 cm in boys and +1.8 cm in girls) SA children had lower subcutaneous fat despite their higher overall fat
Khadgawat Ret al. [27]	Trends in weight gain in children of Indian ethnicity	Boys: BFP increases from age 7 to 11 years, then decreases, reaching a plateau at 14 years Girls: progressive rise in BFP from the age of 7 years until at 17 years it reaches a peak
Ehtisham S et al. [15]	Ethnic differences in body proportions specific to SAs	SAs show larger waist circumference, waist/thigh ratio, and truncal fat compared to those of their Caucasian counterparts Adult gynoid and android body proportions were established as early as 14–16 years of age
Leary S et al., Yajnik CS et al. [28,29]	Thin-fat phenotype	SA neonates had smaller abdominal viscera and lower muscle mass, despite preserved body fat. This phenotype is referred to as the thin-fat phenotype

**Table 3:** Risks of metabolic syndrome in children of SA ethnicity.

References	Findings in SAs
**Insulin Resistance**	
Whincup PH et al. [54]	Insulin resistance is more prevalent among SAs than among Caucasian children, at as early as 10 years of age
Yajnik C S et al. [55]	Hyperinsulinemia, an insulin-resistant phenotype, can be present at birth in SA infants
Ehtisham S et al. [15]	46% of SA adolescents in the UK and 2% of European adolescents have a parent with diabetes 23% of the SA adolescents show signs of insulin resistance; this was not noted in their European counterparts
Sletner L et al. [36]	Prevalence of diabetes mellitus amongst SA adults in the US was found to be 14.3%, which was the highest of level in all other immigrants in the US
**Lipid Abnormalities**	
Vuksan V et al. [56]	62% of SA adolescents had at least one disordered feature of metabolic syndrome whereas this was seen in only 49% of European adolescents
**Cardiovascular Outcomes**	
Gupta R et al. [58,59,60,61]	Prevalence of coronary heart disease is higher amongst SAs, and its manifestation can be seen as much as 10 years earlier than in other ethnic groups [59]
Tillin T et al. [60]	Adverse effects of hyperglycemia on left ventricular function are more severe in SAs [60]
Chahal NS et al. [61]	Prevalence of left ventricular hypertrophy is three times higher and a greater degree of concentric remodeling is evident in SAs compared to British adults even after adjusting for BMI, lean body mass, and height [61]

**Table 4:** Suggestions for clinicians managing SA children with obesity.

Suggestions for Clinicians	
Clinical clues	Pediatric providers must hold a high index of suspicion in SA children with overweight and obesity for: NAFLD Higher inflammatory state Insulin resistance Abnormal lipid profile Sleep breathing disorders Higher blood pressure Future cardiac disorders
Diet	Family-based dietary interventions, especially those that have migrated to western countries: Higher consumption of fruits Less disruption in mealtimes Significant reduction of fast food
Physical Activity	Pediatricians must be cognizant of the role of ethnicity in compliance to exercise recommendations amongst children. SA children display worse adherence to physical activity guidelines in comparison to that of Caucasian children. Emphasis on parental education highlighting the benefits of physical activity in reversing insulin resistance and improving the lipid profile, blood pressure, and cardiovascular health.

**Table 5:** Areas of future research to facilitate management of pediatric obesity in SAs.

Areas for Future Research
Identify a consensus on best alternative to BMI in screening SA children for obesity using insights from methods studied in other ethnicities. Develop an ideal BMI adjustment formula for various pediatric ages in SA boys and girls that better estimates adiposity than does the unadjusted BMI screening tool. More research specific to pediatric populations to determine and understand why SA children harbor higher inflammatory markers and have a higher risk for NAFLD and other obesity-associated metabolic complications similar to those of adults of SA ethnicity.

## Data Availability

Not Applicable.
